# Unveiling risk factors for post-COVID-19 syndrome development in people with type 2 diabetes

**DOI:** 10.3389/fendo.2024.1459171

**Published:** 2024-12-11

**Authors:** Anton Matviichuk, Viktoriia Yerokhovych, Sergii Zemskov, Yeva Ilkiv, Vitalii Gurianov, Zlatoslava Shaienko, Tetyana Falalyeyeva, Oksana Sulaieva, Nazarii Kobyliak

**Affiliations:** ^1^ Department of Endocrinology, Bogomolets National Medical University, Kyiv, Ukraine; ^2^ Department of Endocrinology with Pediatric Infectious Diseases, Poltava State Medical University, Poltava, Ukraine; ^3^ Department of Fundamental Medicine, Educational-Scientific Center “Institute of Biology and Medicine” Taras Shevchenko National University of Kyiv, Kyiv, Ukraine; ^4^ Scientific Department, Medical Laboratory CSD, Kyiv, Ukraine; ^5^ Department of Pathology, Kyiv Medical University, Kyiv, Ukraine

**Keywords:** post-COVID-19 Syndrome, long COVID-19, COVID-19 infection, SARS-CoV-2, type 2 diabetes

## Abstract

**Introduction:**

Post-COVID-19 syndrome (PCS) is a severe acute respiratory syndrome coronavirus 2 (SARS-CoV-2) infection-associated chronic condition characterized by long-term violations of physical and mental health. People with type 2 diabetes (T2D) are at high risk for severe COVID-19 and PCS.

**Aim:**

The current study aimed to define the predictors of PCS development in people with T2D for further planning of preventive measures and improving patient outcomes.

**Materials and methods:**

The data were collected through the national survey targeting persons with T2D concerning the history of COVID-19 course and signs and symptoms that developed during or after COVID-19 and continued for more than 12 weeks and were not explained by an alternative diagnosis. In total, 469 patients from different regions of Ukraine were enrolled in the study. Among them, 227 patients reported PCS development (main group), while 242 patients did not claim PCS symptoms (comparison group). Stepwise multivariate logistic regression and probabilistic neural network (PNN) models were used to select independent risk factors.

**Results:**

Based on the survey data, 8 independent factors associated with the risk of PCS development in T2D patients were selected: newly diagnosed T2D (OR 4.86; 95% CI 2.55–9.28; p<0.001), female sex (OR 1.29; 95% CI 0.86–1.94; p=0.220), COVID-19 severity (OR 1.35 95% CI 1.05–1.70; p=0.018), myocardial infarction (OR 2.42 95% CI 1.26–4.64; p=0.002) and stroke (OR 3.68 95% CI 1.70–7.96; p=0.001) in anamnesis, HbA1c above 9.2% (OR 2.17 95% CI 1.37–3.43; p=0.001), and the use of insulin analogs (OR 2.28 95% CI 1.31–3.94; p=0.003) vs human insulin (OR 0.67 95% CI 0.39–1.15; p=0.146). Although obesity aggravated COVID-19 severity, it did not impact PCS development. In ROC analysis, the 8-factor multilayer perceptron (MLP) model exhibited better performance (AUC 0.808; 95% CІ 0.770–0.843), allowing the prediction of the risk of PCS development with a sensitivity of 71.4%, specificity of 76%, PPV of 73.6% and NPV of 73.9%.

**Conclusions:**

Patients who were newly diagnosed with T2D, had HbA1c above 9.2%, had previous cardiovascular or cerebrovascular events, and had severe COVID-19 associated with mechanical lung ventilation were at high risk for PCS.

## Introduction

Approximately 95% of people with diabetes worldwide have type 2 diabetes (T2D). A 3% increase in age-standardized mortality rates from diabetes was recorded from 2000 to 2019 (1). T2D is a group of metabolic diseases caused by insulin resistance (IR) and altered insulin secretion by β-cells of the pancreas (2, 3).

The 2019 coronavirus disease (COVID-19) pandemic caused by severe acute respiratory syndrome coronavirus 2 (SARS-CoV-2) has become a global concern (4–6). T2D is one of the most common comorbidities in patients infected with the SARS-CoV-2 virus, with a relatively high incidence of severe COVID-19 (7, 8). T2D has a bidirectional relationship with COVID-19 (9). Poorly controlled, decompensated T2D exacerbates the severity of COVID-19 and leads to an increased risk of hospitalization and mortality (10, 11). Potential mechanisms contributing to enhanced susceptibility to SARS-CoV-2 infection and poorer prognosis in people with T2D include a proinflammatory state, weakened innate immune response, possibly elevated levels of angiotensin-converting enzyme 2 (ACE2), vascular dysfunction and a prothrombotic state (12–14). On the other hand, an extreme systemic immune response (“cytokine storm”), direct attack of pancreatic β-cells by SARS-CoV-2 by binding to ACE2, and an unbalanced immune response can, in turn, lead to glycemic profile disorders, uncontrolled hyperglycemia, and progression of IR in persons with T2D (15, 16).

COVID-19 combined with T2D enhances the risk of hospitalization and the need for mechanical ventilation, increasing the probability of post-COVID-19 syndrome development. Post-COVID-19 syndrome (PCS; long COVID-19, post-acute COVID-19, long-term effects of COVID-19) has become an emerging health problem in people recovering from COVID-19 infection (17–19). PCS condition occurs in individuals with a history of probable or confirmed SARS- CoV-2 infection, usually 3 months from the onset of COVID-19 with symptoms that last for at least 2 months and cannot be explained by an alternative diagnosis (20). Common symptoms include rapid fatigue, weakness, headaches, memory loss, distraction, depression, prolonged cough or shortness of breath, insomnia, heart palpitations, bone and joint aches, myalgias, gastrointestinal disorders, and insensitivity to smells and tastes (20). Symptoms may be new onset, following initial recovery from an acute COVID-19 episode or persist from the initial illness. Symptoms may also fluctuate or relapse over time (20).

One of the consequences of lung damage in patients with SARS-CoV-2 infection, namely, pulmonary fibrosis, which can manifest as persistent shortness of breath requiring oxygen supplementation in the PCS period, is more common in people with poorly controlled diabetes (18). Not surprisingly, the bidirectional association between diabetes and PCS has been at the top of scientific discussions (21, 22). Some evidence suggests that diabetes may be a risk factor for the development of PCS (22, 23). Recent data also indicate that new-onset diabetes might be a complication of COVID-19 and represents the metabolic clinical phenotype of PCS (18, 24). However, the particular links between T2D and PCS are still under debate. Limited research exists on PCS incidence and prevalence in low- and middle-income countries.

The current study aimed to define the predictors of PCS development in people with T2D for further planning of preventive measures and improving patient outcomes.

## Materials and methods

### Ethics statement

The study protocol was approved by the Ethics Committee at Bogomolets National Medical University (protocol number: 171/2023) and was conducted according to the guidelines of the 1975 Declaration of Helsinki. Individuals with T2D were enrolled in the study during visits to endocrinologists at outpatient clinics. The purpose and methodology of the study were fully explained to the participants by the researchers, and all patients were asked to provide signed informed consent before data collection.

### Study design

To gather data concerning the outcomes of COVID-19 in T2D persons, a questionnaire was developed. The following clinical and demographic data were collected: age, sex, anthropometric indicators, T2D duration and age at onset, T2D complications, history of COVID-19, COVID-19 severity and treatment, PCS symptoms, duration of PCS and hypoglycemic therapy. According to the WHO classification, COVID-19 was categorized as mild, moderate or severe. Mild COVID-19 was defined as respiratory symptoms without evidence of pneumonia or hypoxia, while moderate or severe infection required the presence of clinical and radiological evidence of pneumonia. In moderate cases, SpO2 ≥90% on room air, while one of the following was required to define severe cases: respiratory rate >30 breaths/min or SpO2 <90% on room air (25, 26). The data were collected and registered by a professional endocrinologist during the follow-up visits of patients to outpatient clinics. Medical data were also retrieved from the medical records of the participants.

The inclusion criteria were as follows: age over 18 years and the presence of T2D and COVID-19 confirmed by a positive RT−PCR test. The exclusion criteria included type 1 diabetes or secondary diabetes, autoimmune diseases, inflammatory diseases, other than T2D metabolic diseases and active malignancy. The data from 469 patients who suffered from COVID-19 infection were collected in different regions of Ukraine. According to the responses, patients were divided into 2 groups depending on the outcomes for up to 6 months after COVID-19 infection: the PCS group (main group, n=227) and patients who didn’t develop PCS (comparison group, n=242).

Body mass index (BMI) was calculated as body weight in kilograms divided by the square of the participant’s height in meters (weight/height^2^). The waist (narrowest diameter between the xiphoid process and iliac crest) circumference (WC) was also measured.

As obesity itself is an immunometabolic disorder, facilitating pro-inflammatory cytokines secretion, reducing insulin sensitivity (27) and modulating SARS-Cov2 retention (28) we provided a sub-analysis for assessing the effect of obesity on PCS development and COVID-19 severity. Patients were divided into two sub-groups including individuals with BMI<30kg/m^2^ (n=110) and patients with obesity (BMI≥30kg/m^2^, n=117).

In addition, cases of new-onset T2D were assessed separately. New-onset T2D in PCS group was defined when occur during or after acute COVID-19 phase within 3 months (n=43). From comparison group we included in sub analysis patients with onset of T2D before 3 months to COVID-19 (n=17).

### Statistical analysis

Statistical analysis was performed using MedCalc^®^ Statistical Software v. 22.026 (MedCalc Software Ltd., Ostend, Belgium; https://www.medcalc.org; 2024) and STATISTICA Neural Networks R.4.0 C (StatSoft. Inc. 1998-1999). To test the normality of the distribution, the Shapiro−Wilk test was used. Quantitative variables are presented as the median and interquartile range (Me, Q_I_ – Q_III_), and qualitative variables are presented as %. To estimate the difference in the incoming qualitative data, the χ^2^ test or Fisher’s exact test was used; for quantitative data, the Mann−Whitney test was used. Univariate logistic regression analysis was applied to assess variables associated with PCS development in patients with T2D.

Stepwise multivariate logistic regression and probabilistic neural network (PNN) models were used to select independent risk factors associated with PCS development. In the first stage, a minimal set of variables associated with PCS risk was selected. To select independent risk factors for multivariate logistic regression models, stepwise inclusion/exclusion of variables (stepwise with p_enter_ <0.1 та p_remove_>0.2) was performed, and the genetic algorithm (GA) method of selection was used for the PNN models. For the PNN models, all patients were randomly (using a random number generator) divided into 3 sets: training (which was used to build the model and calculate weight coefficients of the neural network, n=369), test (used to prevent overtraining of the mathematical model, n=60) and verification (used to test the predictive ability of the mathematical model on new data for controlling model retraining, n=40) sets.

The diagnostic performance of the models was evaluated using receiver operating characteristic (ROC) curve analysis. The area under the ROC curve (AUC) and its 95% confidence interval (CI) were calculated. A p value < 0.05 was considered to indicate statistical significance in all tests. Optimal cutoff values were chosen to maximize the sum of sensitivity and specificity. Positive predictive values (PPVs) and negative predictive values (NPVs) were computed for these cutoff values (29).

## Results

### Patient characteristics

The baseline clinical parameters, COVID-19 and T2D histories of the surveyed patients are presented in **Table 1**. Among the study subjects, the ages in the main group were 61 (54 – 67) and 60 (54 – 68) years (p=0.900), respectively. The main group comprised more patients over 60 years old (59% vs 53.7% in the comparison group), although these differences were not significant (p=0.264) (**Table 1**). There were 124 females (54.6%) in the PCS group, while the proportion of women was lower among patients with no PCS (115 out of 242, 47,5); however, sex differences were not significant between the groups (p=0.139). We also did not find differences in patient weight (p=0.994) or BMI (p=0.881) (**Table 1**).

**Table 1:** Baseline clinical parameters and COVID-19 and T2D history in surveyed patients.

Parameter	Comparison group (no PCS) (n=242)	Main group (PCS) (n=227)	p
Age, years	60 (54 – 68)	61 (54 – 67)	0.900
Age over 60 years, n (%)	130 (53.7)	134 (59)	0.264
Females, n (%)	115 (47.5)	124 (54.6)	0.139
T2D duration, years	11 (7 – 16)	10 (3 – 15)	0.015
Newly diagnosed diabetes, n (%)	17 (7.0)	43 (18.9)	<0.001
Weight, kg	88 (80 – 98)	88 (78 – 100)	0.994
Height, cm	169 (165 – 176)	168 (163 – 177)	0.384
BMI, kg/m^2^	29.8 (27.500 – 33.8)	30.7 (26.925 – 34)	0.881
HbA1C before, %	7.9 (7 – 9)	8.2 (7.2 – 10)	0.005
Poor glycemic control (HbA1c>7.5), n (%)	154 (63.6)	160 (70.5)	0.118
T2D chronic complication			
Diabetic nephropathy, n (%)	71 (29.3)	54 (23.8)	0.210
Diabetic neuropathy, n (%)	160 (66.1)	147 (64.8)	0.771
Diabetic retinopathy, n (%)	119 (49.2)	98 (43.2)	0.196
Diabetic foot, n (%)	43 (17.8)	26 (11.5)	0.067
Myocardial infarction, n (%)	19 (7.9)	31 (13.7)	0.051
Stroke, n (%)	11 (4.5)	27 (11.9)	0.004
No complication, n (%)	51 (21.1)	60 (26.4)	0.120
T2D treatment			
No medical treatment, n (%)	8 (3.3)	18 (7.9)	0.042
Metformin, n (%)	161 (66.5)	156 (68.7)	0.623
Sulfonylureas, n (%)	86 (35.5)	68 (30)	0.203
DPP-4 inhibitors, n (%)	20 (8.3)	10 (4.4)	0.093
GLP-1 agonists, n (%)	9 (3.7)	13 (5.7)	0.383
SGLT-2 antagonists, n (%)	21 (8.7)	33 (14.5)	0.059
PPAR-γ agonists, n (%)	3 (1.2)	0 (0)	0.249
Human insulin, n (%)	58 (24)	36 (15.9)	0.029
Insulin analogs, n (%)	30 (12.4)	53 (23.3)	0.002
COVID-19 history			
COVID-19 severity (WHO), n (%) Mild Moderate without hospitalization Moderate with hospitalization Severe	107 (44.2) 91 (37.6) 40 (16.5) 4 (1.7)	85 (37.4) 55 (24.2) 65 (28.6) 22 (9.7)	<0.001
No treatment, n (%)	23 (9.5)	14 (6.2)	0.230
Supplements/NSAIDs, n (%)	203 (83.9)	190 (83.7)	0.999
Antibiotics, n (%)	157 (64.9)	152 (67)	0.697
O_2_ therapy, n (%)	61 (25.2)	71 (31.3)	0.152
Steroids, n (%)	61 (25.2)	97 (42.7)	<0.001
Mechanical ventilation, n (%)	1 (0.4)	16 (7)	<0.001

The data are presented as the Me (Q_I_ - Q_III_) or n (%); BMI, body mass index; NSAIDs, nonsteroidal anti-inflammatory drugs; WHO, World Health Organization; PCS, post-COVID-19 syndrome. Bold values indicate significant changes.

**Figure 1:**
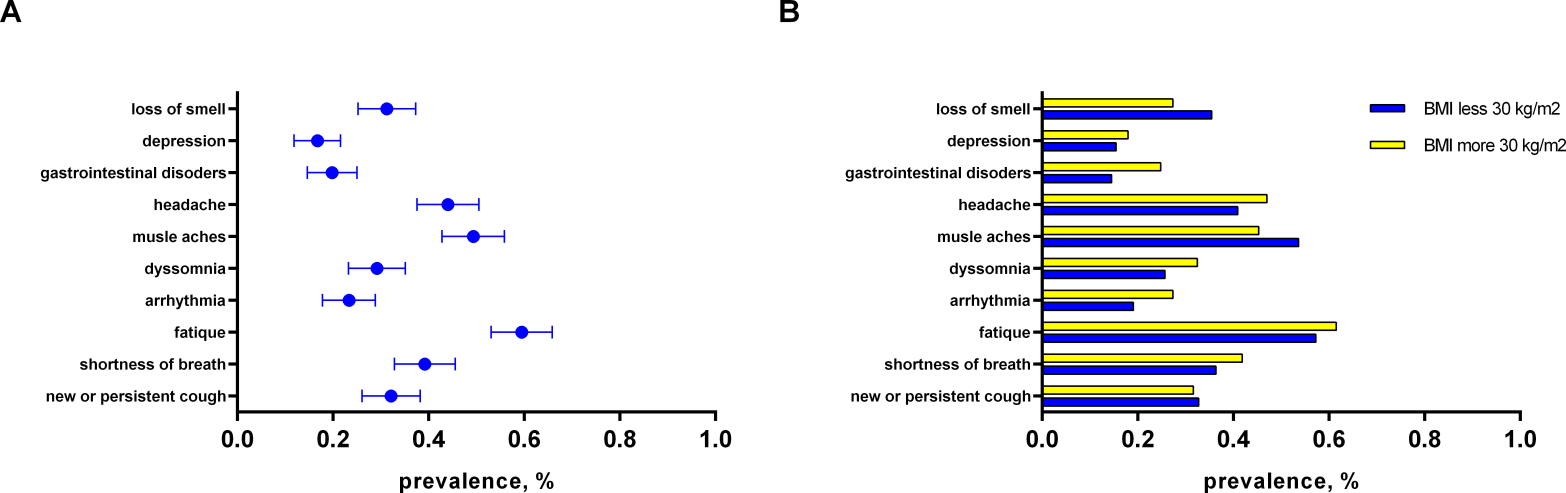
The distribution of PCS symptoms: **(A)** among persons with T2D (main group); **(B)** sub analysis depending on presence of obesity in patients among PCS group.

By assessing the clinical phenotypes of PCS among T2D patients, we found that fatigue was the most often observed manifestation of PCS (59.5%). It was followed by muscle aches (49.3%), headache (44.1%), shortness of breath (39.2%), new or persistent cough (31.7%), loss or change of smell (31.3%), dyssomnia (28.8%), arrhythmia (23.3%), gastrointestinal disorders (19.8%), and depression (16.7%) (**Figure 1A**).

### Medical history of diabetes and beyond – the links to PCS

The subjects who suffered from PCS reported a medical history of poor glycemic control during anamnesis and had higher values of HbA1c than did those in the comparison group (8.2% (7.2 - 10) vs 7.9% (7 – 9); p=0.005) (**Table 1**). Surprisingly, the mean duration of T2D was lower in the main group 10 (3–15) years as compared to the comparison group - 11 (7 – 16) years (p=0.015) (**Table 1**). This finding was explained by the increased incidence of newly diagnosed T2D during the COVID-19 pandemic, representing one of the clinical phenotypes of PCS. The number of patients with newly developed T2D was 43 out of 224 patients in the main group (18.9%), while only 17 individuals with newly diagnosed T2D were identified in the comparison group (7.0%; p<0.001) (**Table 1**). In sub analysis patient with new-onset T2D from PCS group characterized with more aggressive presentation as compared to matched patient from comparison group (7.0 (6.5 – 8.8) vs 7.8% (6.5 – 9.4); p=0.344). We did not find significant differences in the age of patients with new T2D between groups (57.0 (47.5 – 69.0) vs 58.0 (45.0 – 64.0) years, p=0.582). Surprisingly, patients with new-onset T2D in PCS group had lower weight (88.0 (80.0 – 96.0) vs 97.0 (85.0 – 109.0) kg; p=0.043) and BMI (28.4 (25.2 – 31.2) vs 31.5 (28.4 – 38.1) kg/m^2^; p=0.021) as compared to those with no PCS.

It is also worth noting that patients with PCS had a greater incidence of diabetic macrovascular complications: 13.7% and 11.9% of PCS patients reported myocardial infarction and stroke, respectively, during anamnesis (**Table 1**). In patients without PCS, a significantly lower prevalence of cardiovascular events was reported in their medical history: 7.9% for myocardial infarction (p=0.051) and 4.5% for stroke (p=0.004). The incidence of microvascular T2D complications did not differ significantly between the groups (**Table 1**).

PCS was associated with a greater rate of hospitalization and a more severe COVID-19 course. By comparing the COVID-19 course, we found that in the comparison group, most patients demonstrated mild (107; 44.2%) or moderate COVID-19 without hospitalization (91; 37.6%), and only a relatively small portion of the group had a moderate course of hospitalization (40; 16.5%) or suffered from severe COVID-19 (4; 1.7%) (**Table 1**). In contrast, approximately 40% of the main group reported severe (22; 9.7%) or moderate disease, with hospitalization (65; 28.6%) impacting further PCS development (p<0.001) (**Table 1**).

### To what extent can treatment affect the risk of PCS?

Importantly, PCS development was associated with a higher rate of noncompliance with antidiabetic medications: 7.9% of patients in the main group didn’t follow treatment recommendations, while the percentages were less than half in the comparison group (3.3%; p=0.042) (**Table 1**). We also found a difference in the rate of insulin and its analog administration between the observed groups: patients without PCS were more often administered human insulin than were those in the main group (24% vs 15.9%; p=0.029), where insulin analogs were used more often (23.3% vs 12.4%; p=0.002) (**Table 1**). In terms of the treatment of T2D, we did not find significant differences among the prescribed anti-diabetic drugs (ADDs) between the groups (**Table 1**).

There were also peculiarities related to COVID-19 treatment. Considering the increased rate of hospital admission and severe COVID-19 history, the PCS group reported a significantly increased rate of steroid prescription (42.7% vs 25.2%; p<0.001) and mechanical ventilation (7% vs 0.4%; p<0.001) due to the severity of COVID-19. At the same time, we did not find differences in the prescription of NSAIDs, antibiotics or O_2_ therapy between the groups (**Table 1**).

### Is there a link between obesity and PCS?

Obese people with T2D were more frequently diagnosed with moderate and severe forms of COVID-19 infection as compared to patients with BMI<30 (46.1% vs 30.0%, p=0.011; **Table 2**). Besides, glucocorticoid prescription was more frequent in obese patients with T2D (53.8% vs 30.9%; p<0.001; **Table 2**) during the COVID-19 course. The data representing clinical parameters, COVID-19 and T2D history in PCS group with respect to obesity are presented in **Table 2**. Important that T2D duration in normal/overweight patients was significantly shorter as compared to obese (8.5 (2.8 – 14.0) vs 11.0 (5.0 – 17.0) years, p=0.013). This finding can be explained by the higher occurrence of newly onset T2D in PCS group (**Table 2**). The profile of T2D complication and anti-diabetic treatment didn`t differ significantly between subgroups (**Table 2**). Moreover, there were no significant differences in PCS symptoms in T2D patients regarding obesity (**Figure 1B**).

**Table 2:** The sub analysis for distribution of clinical parameters, COVID-19 and T2D history depending on presence of obesity in patients of PCS group.

Parameter	BMI ≤ 30 kg/m^2^ (n=110)	BMI>30 kg/m^2^ (n=117)	p
Age, years	61.0 (53.0 – 66.3)	62.0 (55.0 – 68.0)	0.308
Age over 60 years, n (%)	61 (55.5)	73 (62.4)	0.288
Females, n (%)	63 (57.3)	61 (52.1)	0.437
T2D duration, years	8.5 (2.8 – 14.0)	11.0 (5.0 – 17.0)	0.013
Newly diagnosed diabetes, n (%)	27 (24.5)	16 (13.7)	0.037
Weight, kg	78.0 (68.0 – 87.0)	99.0 (89.5 – 110.0)	<0.001
Height, cm	172.0 (165.0 – 179.0)	167.0 (162.5 – 176.0)	0.042
BMI, kg/m^2^	26.9 (24.0 – 28.0)	33.7 (32.0 – 37.1)	<0.001
HbA1C before, %	8.2 (7.2 – 10.0)	8.3 (7.2 – 10.0)	0.991
T2D chronic complication			
Diabetic nephropathy, n (%)	24 (21.8)	30 (25.6)	0.499
Diabetic neuropathy, n (%)	65 (59.1)	82 (70.1)	0.083
Diabetic retinopathy, n (%)	45 (40.9)	53 (45.3)	0.505
Diabetic foot, n (%)	11 (10.0)	15 (12.8)	0.505
Myocardial infarction, n (%)	13 (11.8)	18 (15.4)	0.434
Stroke, n (%)	16 (14.5)	11 (9.4)	0.232
No complication, n (%)	31 (28.2)	29 (24.8)	0.562
T2D treatment			
No medical treatment, n (%)	9 (8.2)	9 (7.7)	0.891
Metformin, n (%)	68 (61.8)	87 (74.4)	0.090
Sulfonylureas, n (%)	31 (28.2)	37 (31.6)	0.572
DPP-4 inhibitors, n (%)	5 (4.5)	5 (4.3)	0.921
GLP-1 agonists, n (%)	3 (2.7)	10 (8.5)	0.059
SGLT-2 antagonists, n (%)	18 (16.4)	15 (12.8)	0.449
Human insulin, n (%)	14 (12.7)	22 (18.8)	0.210
Insulin analogs, n (%)	31 (28.2)	22 (18.8)	0.095
COVID-19 history			
COVID-19 severity (WHO), n (%) Mild Moderate without hospitalization Moderate with hospitalization Severe	51 (46.4) 26 (23.6) 28 (25.5) 5 (4.5)	34 (29.1) 29 (24.8) 37 (31.6) 17 (14.5)	0.011
No treatment, n (%)	8 (7.3)	6 (5.1)	0.488
Supplements/NSAIDs, n (%)	94 (85.5)	96 (82.1)	0.502
Antibiotics, n (%)	70 (63.6)	82 (70.1)	0.302
O_2_ therapy, n (%)	28 (25.5)	43 (36.8)	0.067
Steroids, n (%)	34 (30.9)	63 (53.8)	<0.001
Mechanical ventilation, n (%)	2 (1.8)	4 (3.4)	0.452

The data are presented as the Me (Q_I_ - Q_III_) or n (%); BMI, body mass index; NSAIDs, nonsteroidal anti-inflammatory drugs; WHO, World Health Organization; PCS, post-COVID-19 syndrome. Bold values indicate significant changes.

It is worth noting that patients with and without PCS did not differ in BMI (**Table 1**). Similarly, there were no differences in the incidence of obesity between PCS and comparison groups. The shares of people with obesity comprised 51.5% (117 of 227) among the PCS group being comparable with the value in the comparison group (116 of 242; 47.8%; p=0.435). Finally, among 233 T2D patients with comorbid obesity, about half (117; 50.2%) reported PCS symptoms while the rest – no, demonstrating no impact of obesity on PCS development in the observed cohort.

Thus, comorbid obesity aggravated COVID-19 severity but did not impact PCS development in patients with T2D.

### Uncovering the prognostic factors contributing to PCS

Univariate logistic regression analysis revealed the following independent predictors of PCS development in patients with T2D: newly diagnosed T2D (p<0.001), poor glycemic control with an HbA1c above 9.2% (p<0.001), history of myocardial infarction (p=0.044) or stroke (p=0.005), treatment of T2D with insulin analogs (p=0.002), moderate-to-severe COVID-19 course (p<0.001), history of treating COVID-19 with glucocorticoids (p<0.001) and mechanical ventilation (p=0.005). In contrast, the use of human insulin (OR 0.598; 95% CI 0.377-0.950; p=0.029) had a protective effect on PCS development (**Table 3**).

**Table 3:** Univariate logistic regression analysis.

Variables		Event/Total	Odds ratio	95% CI	p
Diab foot	No	201/400	0.599	0.354-1.012	0.055
	Yes	26/69			
Diabetic nephropathy	No	173/344	0.752	0.498-1.135	0.175
	Yes	54/125			
Diabetic retinopathy	No	129/252	0.785	0.546-1.130	0.193
	Yes	98/217			
Diabetic neuropathy	No	80/162	0.942	0.643-1.378	0.757
	Yes	147/307			
Age	<=60 Y	93/205	1.241	0.861-1.790	0.247
	>60 Y	134/264			
T2D duration	Long-term	184/409	3.093	1.707-5.604	<0.001
	New onset	43/60			
Gender	Male	103/230	1.33	0.925-1.912	0.124
	Female	124/239			
No complication	No	167/358	1.346	0.878-2.063	0.173
	Yes	60/111			
Myocardial infarction	No	196/419	1.856	1.016-3.391	0.044
	Yes	31/50			
HbA1c	>9.2	148/342	2.157	1.421-3.276	<0.001
	<=9.2	79/127			
Stroke	No	200/431	2.835	1.371-5.860	0.005
	Yes	27/38			
T2D treatment					
PPAR-γ agonists	No	227/466	0.15	0.0077-2.928	0.191
	Yes	0/3			
DPP-4 inhibitors	No	217/439	0.512	0.234-1.118	0.093
	Yes	10/30			
Human insulin	No	191/375	0.598	0.377-0.950	0.029
	Yes	36/94			
Sulfonylureas	No	159/315	0.776	0.527-1.143	0.199
	Yes	68/154			
Metformin	No	71/152	1.098	0.745-1.618	0.635
	Yes	155/316			
GLP-1 agonists	No	214/447	1.573	0.659-3.754	0.308
	Yes	13/22			
SGLT-2 inhibitors	No	194/415	1.79	1.002-3.198	0.051
	Yes	33/54			
Insulin analogs	No	174/386	2.152	1.318-3.516	0.002
	Yes	53/83			
No treatment	No	209/443	2.519	1.073-5.914	0.034
	Yes	18/26			
COVID-19 history					
No treatment	No	213/432	0.626	0.314-1.249	0.184
	Yes	14/37			
Supplements/NSAIDs	No	37/76	0.987	0.604-1.613	0.957
	Yes	190/393			
Antibiotics	No	75/160	1.097	0.749-1.608	0.476
	Yes	152/309			
O_2_ therapy	No	156/337	1.35	0.902-2.022	0.145
	Yes	71/132			
Steroids	No	130/311	2.214	1.497-3.275	<0.001
	Yes	97/158			
COVID severity	<=2	140/338	2.796	1.833-4.266	<0.001
	>2	87/131			
Mechanical ventilation	No	211/452	18.275	2.403-138.973	0.005
	Yes	16/17			

Bold values indicate significant changes.

**Figure 2:**
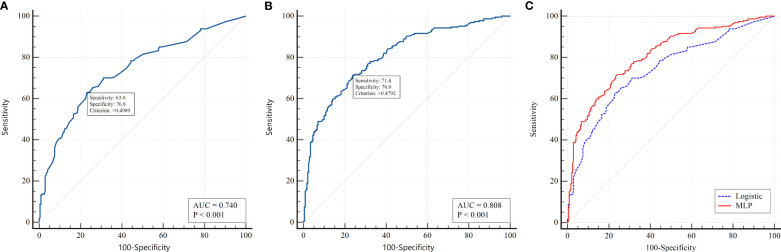
ROC analysis for predicting PCS in patients with T2D. **(A)** logistic regression model; **(B)** MLP model; **(C)** pairwise comparison between models.

To select the most informative risk factors, multifactorial logistic regression analysis was applied. As a result of the selection, the following 8 independent factors associated with the risk of PCS development in T2D patients were selected: newly diagnosed T2D (OR 4.86; 95% CI 2.55 – 9.28; p<0.001), female sex (OR 1.29; 95% CI 0.86 – 1.94; p=0.220), COVID-19 severity (OR 1.35 95% CI 1.05 – 1.70; p=0.018), presence of myocardial infarction (OR 2.42 95% CI 1.26 – 4.64; p=0.002) and stroke (OR 3.68 95% CI 1.70 – 7.96; p=0.001) in anamnesis, HbA1c above 9.2% (OR 2.17 95% CI 1.37 – 3.43; p=0.001), use of insulin analogs (OR 2.28 95% CI 1.31 – 3.94; p=0.003) vs human insulin (OR 0.67 95% CI 0.39 – 1.15; p=0.146), as specified in **Table 4**. The AUROC of the model was 0.74 (95% CI 0.697 - 0.779; p<0.001) (**Figure 2A**). This model demonstrated modest accuracy, as presented in **Table 5**.

**Table 4:** Coefficients of the 8-factor logistic regression model for PCS risk prediction.

Factor		b ± m	p	OR (95% CI)
COVID severity		0.29 ± 0.12	0.018	1.35 (1.05 – 1.70)
Mechanical ventilation		2.85 ± 1.08	0.008	17.4 (2.11 – 143)
Myocardial infarction		0.88 ± 0.33	0.008	2.42 (1.26 – 4.64)
Gender (female vs male)		0.25 ± 0.21	0.220	1.29 (0.86 – 1.94)
Stroke		1.30 ± 0.39	0.001	3.68 (1.70 – 7.96)
HbA1c (>9.2 vs<=9.2)		0.77 ± 0.23	0.001	2.17 (1.37 – 3.43)
T2D duration	Long-term	Reference		
	New onset	1.58 ± 0.33	<0.001	4.86 (2.55 – 9.28)
Insulin	No insulin	Reference		
	Human insulin	-0.40 ± 0.28	0.146	0.67 (0.39 – 1.15)
	Insulin analogs	0.82 ± 0.28	0.003	2.28 (1.31 – 3.94)

**Table 5:** Diagnostic accuracy of the proposed models for predicting PCS.

Parameter	8-factors logistic	8-factors MLP
Cutoff value	>0.4989	>0.4792
Sensitivity, % (95% CI)	63.0 (56.4 - 69.3)	71.4 (65.0 - 77.2)
Specificity, % (95% CI)	76.8 (70.9 - 81.9)	76.0 (70.1 - 81.3)
NPV, % (95% CI)	68.8 (64.7 - 72.6)	73.9 (69.5 - 77.9)
PPV, % (95% CI)	71.9 (66.5 - 76.6)	73.6 (68.7 - 78.0)
-LR, (95% CI)	0.48 (0.40 - 0.58)	0.38 (0.30 - 0.47)
+LR, % (95% CI)	2.71 (2.11 - 3.48)	2.98 (2.34 - 3.78)
AUC	0.740	0.808
95% CІ	0.697 - 0.779	0.770 - 0.843
p (AUC)	<0.001	<0.001

NPV, negative predictive value; РPV, positive predictive value; LR, likelihood ratio; AUC, area under the ROC curve; 95% CІ, 95% confidence interval for the AUC.

In the second stage, we built PNN models based on nonlinear relationships between variables and outcomes. We used a multilayer perceptron (MLP) with one hidden layer. The architecture of the hidden layer had 3 neurons with a logistic activation function. According to the ROC analysis, the AUC for the MLP model was 0.808 (95% CІ 0.770 - 0.843, p<0.001) (**Figure 2B**). The cutoff value for this model was chosen based on the Youden index (>0.490). When applying the optimal threshold, the following characteristics of the model were detected: sensitivity, 71.4% (95% CІ 65.0 - 77.2%); specificity, 76.0% (95% CІ 70.1 - 81.3%); PPV, 73.6% (95% CІ 68.7 - 78.0%); and NPV, 73.9% (95% CІ 69.5 - 77.9%) (**Table 5**). The forecasting results using neural networks were significantly better than those of the logistic model (p<0.001). The results of pairwise comparisons of the ROC curves are presented in **Figure 2C**. This indicates the presence of nonlinearity in the relationship between PCS risk and factor attributes that cannot be taken into account in a simple regression model.

## Discussion

Although high BMI and diabetes have been recognized as risk factors for developing severe COVID-19 and PCS, there are still no clearly articulated predictors of PCS development in T2D patients who restrict preventive measures for improving patient outcomes and quality of life (30). This study revealed the key risk factors associated with the risk of PCS development in T2D patients.

By applying various types of logistic regression analysis and PNN, we identified risk factors, including female sex, COVID-19 severity and corresponding mechanical ventilation experience, newly diagnosed during COVID-19 diabetes and an HbA1c higher than 9.2%, as well as myocardial infarction or stroke in medical history, as key risk factors for PCS prediction. Various studies of PCS prognosis have also revealed the prognostic role of different factors. Despite the variability of the results, the ability of core factors, including female sex and COVID-19 severity, to predict PCS has been underscored in different studies. Maglietta et al. in systematic review demonstrated the role of female sex and acute disease severity (31). Lemhöfer et al. reported that female sex, preexisting coagulation disorders and coronary artery disease were associated with a higher PCS rate (32). Similarly, in multivariate analysis, Zemni et al. showed that female sex, preexisting comorbidities, duration of acute COVID-19 illness, hospitalization, number of COVID-19 episodes and vaccination against SARS-CoV-2 are important in defining the risk of PCS development (33). These data obtained from the whole population analysis are consistent with our findings supporting the role of sex and acute viral infection severity. In this study, involving exclusively T2D patients, additional factors, including T2D severity and insulin treatment, were found to be essential for estimating the probability of PSC.

Diabetes is associated with a high risk of adverse outcomes of COVID-19 infection and PCS (30). On the other hand, the current study did not confirm that the presence of T2D was a risk factor for long-term symptoms of PCS (34). According to the obtained results, patients with and without T2D who recovered from COVID-19 at 7.2 (SD 0.6) months after hospital discharge had similar incidence rates of PCS symptoms (1.06, 95% CI 0.92-1.24; p=0.372) (34). Therefore, additional factors influencing the outcome of COVID-19 infection should be investigated.

Another challenge which is actively debated is assessment of preadmission use of different ADD for on COVID-19 adverse outcomes, mortality as well PCS development and severity. Recent studies revealed that pretreatment with metformin, GLP-1RA, and SGLT-2i was associated with a lower mortality rate, main adverse outcomes and hospitalization in patients with COVID-19 and T2D (35, 36). DPP-4i use was associated with a statistically significant increase in the risk of hospitalization, admission (35) to the ICU and mortality (36). Treatment with insulin is a risk factor for hospitalization and increased mortality (36, 37). The effects of sulfonylurea, thiazolidinedione, and alpha-glucosidase inhibitors on mortality are neutral (36). In contrast, the current study did not find a significant association between at-home ADD administration and mortality or adverse outcomes in patients with T2D admitted for COVID-19 (38). Recent data regarding the association between common T2D treatments and PCS development are scarce and limited to several reports on the protective effects of metformin (39–41). For instance, a recent multicenter, randomized, quadruple-blind, parallel-group, phase 3 trial demonstrated that outpatient treatment with metformin reduced the PCS incidence by approximately 41% (42). In our study, we noticed that insulin analogs significantly increased the risk of PCS development; in contrast, the use of human insulin had a protective effect. The other types of ADDs were neutral.

A close association between T2D and COVID-19 emerged early during the pandemic and is still active (43–45). A history of diabetes in subjects with acute SARS-CoV-2 infections was shown to worsen all outcomes and increase mortality (46). Although the respiratory system is the primary target of SARS-CoV-2, many other organs and cells can be affected by the virus, including the endothelium, cardiomyocytes, immune cells and β-cells of the pancreas. In fact, diabetes and SARS-CoV-2 infection share two essential commonalities - inflammatory pathway activation and multiorgan involvement in pathological processes (47, 48). This can result in manifestations of various severe pathologies, including acute cardiovascular dysfunction, digestive system disorders, neurological complications and metabolic disturbances.

Both severe COVID-19 and inefficient glucose control aggravated PCS development in T2D patients. However, there is a bidirectional interplay between COVID-19 and T2D (43), establishing a vicious cycle that facilitates the development of complications (49). SARS-CoV-2 infection and diabetes share two fundamental features: an inflammatory state and multiorgan involvement and damage (50). Notably, the close relationship between immunity and the endocrine system impacts immune cell functionality and the response to viruses. For instance, insulin can directly regulate immune cells, including T-lymphocytes, which are responsible for antiviral immunity (51). Both CD4+ and CD8+ T cells express insulin receptors, which are involved in facilitating glucose uptake and promoting glycolytic metabolism during T-cell activation (52). Moreover, an acute decrease in insulin levels impairs CD8+ T-cell responses to infection, whereas the injection of basal insulin increases the antiviral potential of these cells (53). Hyperglycemia directly undermines the key function of immune cells (54). High blood glucose is related to impaired cytotoxicity of CD8+ and NK cells, as well as abnormal cytokine production by CD4+ T cells, in patients with T2D following infection (55). In addition, HbA1c was shown to positively correlate with the course of infections induced by different pathogens, impacting both disease duration and severity (51). This mechanism could be related to the stimulatory effect of hyperglycemia on the replication of several pathogens (54), impeding the ability of the immune system to fight infectious agents. Thus, our findings showed that poor glycemic control or the use of insulin analogs instead of human insulin increased the risk of PCS development in T2D patients after COVID-19.

We also found that a history of myocardial infarction or stroke can significantly increase the risk of PCS. This finding is consistent with previous findings demonstrating the impact of coronary artery disease on increasing the rate of PCS. Similarly, cardiovascular comorbidities and cerebrovascular events were also shown to enhance the probability of PCS (56). Both myocardial infarction and stroke have common mechanisms in their pathogenesis based on the compromised regulation of the blood clotting system (platelet aggregation and coagulation cascades), endothelial dysfunction, mild long-term inflammation and oxidative stress (57). Importantly, most of these pathophysiological processes interact with theories about the mechanisms underlying PCS development. In addition to persisting viral reservoirs and sustained inflammation, with autoimmune components, dysfunction of the endothelium and corresponding alterations in blood clotting have been underscored in PCS pathogenesis (58, 59). Notably, endothelial dysfunction, inflammation and blood clotting are closely related to myocardial infarction and stroke. These mechanisms are also main players in diabetic progression and complication development. Therefore, patients who experience myocardial infarction and stroke are at high risk for PCS and should be considered for preventing PCS complications. Thus, patients who experience severe COVID-19, especially those on mechanical ventilation, are at greater risk for long-term PCS, which can affect both their physical and mental health.

Finally, we found that patients with T2D manifested during acute COVID-19 infections more frequently observed in normal/overweight persons and characterized with more aggressive presentation as compared to matched patient with onset of T2D before COVID-19. This finding addresses recent scientific discussions of a new specific type of diabetes. It’s still debatable if this phenomenon represents abrupt onset of classical type 1 and type 2 diabetes or a new type of diabetes? Preliminary studies have provided evidence that β-cell infection may be involved in COVID-19 pathogenesis or, alternatively, that pancreatic infection may impact β cells by changing their local microenvironment. The precise underlying mechanisms are not clearly defined, the existing research studies suggests that the pathogenesis of new-onset diabetes due to COVID-19 might be linked to direct viral effects on pancreatic islets as well as systemic inflammatory responses that disrupt glucose metabolism (60). SARSCoV-2 uses ACE2 to enter human cells and TMPRSS2 for ‘priming’ (61). Both proteins are highly expressed in gastrointestinal epithelial cells, pancreatic ductal, acinar and islet cells (62). SARS-CoV-2 is also able to cause diffuse severe endotheliitis of the submucosal vessels in several anatomical sites, and these changes, in turn, cause diffuse microischemic disease (63). Similar ischemic damage could occur in the pancreas due to expression of ACE2 isoform in pancreatic microvasculature (64). Infected pancreatic islets demonstrated reduced glucose-stimulated insulin secretion, fewer insulin granules (65, 66) and characterized with increased islet-cell apoptosis hat may be due to the viral spike protein (60). Müller et al. also suggested that infected cells may lose their hormone content via de-differentiation (65). Data from current studies at least partly could give the background for more aggressive presentation of T2D obtained by our results.

### Limitations

The study was based on questionnaire results that can impact the accuracy of the data and details concerning treatment regimens. When recording the COVID-19 history, there were no data documenting the test systems used for the diagnostics, viral load and vaccination against SARS-CoV-2.

## Conclusion

This study revealed several risk factors facilitating PCS development in T2D patients in Ukraine. We found that patients who were newly diagnosed with T2D, had an HbA1c above 9.2%, had previous cardiovascular or cerebrovascular events, and had severe COVID-19 associated with mechanical lung ventilation were at high risk for PCS development. The developed predictive PNN model allows us to assess the probability of PCS in T2D patients and identify high-risk groups for tailoring their treatment during viral infection.

New-onset T2D which occur during or after acute COVID-19 phase more frequently observed in normal/overweight persons and characterized with more aggressive presentation as compared to matched patient with onset of T2D before COVID-19.

## Data Availability

The raw data supporting the conclusions of this article will be made available by the authors, without undue reservation.
